# CNN-RNN framework for lung cancer classification using CT imaging and GAN-based augmentation

**DOI:** 10.3389/frai.2026.1813190

**Published:** 2026-07-28

**Authors:** Bodicherla Siva Sankar, D. Natarajasivan, M. Purushotham Reddy

**Affiliations:** 1 Department of Computer Science and Engineering, Faculty of Engineering and Technology, Annamalai University, Chidambaram, Tamil Nadu, India; 2 Department of Computer Science and Engineering, Koneru Lakshmaiah Education Foundation, Bowrampet, Hyderabad, Telangana, India

**Keywords:** CNN–RNN framework, CT imaging, deep learning, explainable AI, GAN-based augmentation, Grad-CAM, LSTM networks, lung cancer classification

## Abstract

Lung cancer remains one of the leading causes of cancer-related deaths worldwide, and early identification of malignant abnormalities plays an important role in improving patient survival rates. However, accurate lung cancer classification using CT imaging remains challenging because of limited dataset availability, class imbalance, overlapping lesion characteristics, and lack of interpretability in existing deep learning systems. This study presents a GenAI-driven CNN–RNN framework for explainable lung cancer classification using CT imaging and GAN-based augmentation. The proposed framework integrates convolutional neural networks for spatial feature extraction, LSTM-based recurrent learning for sequential dependency analysis, GAN-assisted augmentation for improving minority class representation, and attention-guided feature fusion for enhanced classification performance. The experimental evaluation was conducted using the publicly available IQ-OTH/NCCD lung cancer CT imaging dataset containing Normal, Benign, and Malignant categories. During preprocessing, normalization, resizing, and image enhancement operations were applied to improve image consistency before training. The framework was trained using the Adam optimizer with 50 epochs and evaluated using 5-fold cross-validation. Experimental results demonstrated that the proposed framework achieved an accuracy of 92.84%, precision of 91.76%, recall of 90.42%, F1-score of 91.08%, and ROC-AUC value of 0.94. Grad-CAM and SHAP visualization methods further improved interpretability by highlighting important lesion regions influencing prediction outcomes. The obtained findings suggest that the proposed CNN–RNN framework can support CT image-based lung cancer classification under limited dataset conditions for CT image-based lung cancer classification under limited medical imaging conditions.

## 1 Introduction

### 1.1 Background and motivation

Lung cancer is still one of the most life-threatening forms of cancer globally, with millions of lives lost each year due to its rapid progression and typically late diagnosis (Javed et al., 2024; Sun et al., 2024). Early and accurate detection is vital for increasing survival chances and improving treatment outcomes. However, conventional diagnostic methods—such as CT scans reviewed by radiologists—often fall short in terms of reliability, scalability, and speed, especially in healthcare systems with limited resources (Patel and Srinivasan, 2025; Quanyang et al., 2024). With the rise of Artificial Intelligence (AI), particularly Deep Learning (DL), medical diagnostics have undergone a major transformation. AI-powered tools now offer automated solutions for lung cancer detection with promising accuracy (Gayap and Akhloufi, 2024; Mathew et al., 2020). Convolutional Neural Networks (CNNs), in particular, have shown strong capabilities in extracting spatial features from CT images (Klangbunrueang et al., 2025; Thanoon et al., 2023). Yet, they are not well-equipped to model complex feature dependencies extracted from CT images. To address this, Recurrent Neural Networks (RNNs), and especially their LSTM variants, are used to understand temporal dependencies and textual data more effectively (Aharonu and Ramasamy, 2024; Crasta et al., 2024).

### 1.2 Challenges in existing deep learning methods

Despite these advancements, real-world application of deep learning in lung cancer diagnosis still faces major challenges. One of the biggest issues is the limited availability of well-labeled malignant cases, which leads to data scarcity during training (Çay, 2024; Elham et al., 2024). This often causes overfitting, where the model performs well on the training set but fails to generalize to new, unseen data (Tang et al., 2024). The result is a generalization gap that hinders clinical reliability and robustness (Musthafa et al., 2024). Another key problem is the lack of interpretability. Deep learning models are often criticized for being “black boxes,” making it difficult for clinicians to trust their predictions without understanding how decisions are made (Zhou and Xin, 2022). As a result, integrating explainable AI (XAI) has become essential for building transparent and trustworthy diagnostic tools.

### 1.3 The unified GenAI-based CNN–RNN solution

To tackle these challenges, this study proposes a unified AI framework that combines the strengths of CNNs and RNNs with the power of Generative AI. Our model aims to classify lung cancer cases into three categories—Benign, Malignant, and Normal—by using CNNs for spatial analysis and RNNs for understanding sequential or textual information (Wei et al., 2025; Rehman et al., 2024). A key feature of our approach is the use of Generative Adversarial Networks (GANs) for synthetic data augmentation. These networks create realistic artificial samples that help fill in gaps in the original dataset-especially underrepresented malignant cases—leading to improved model generalization (Çay, 2024; Aharonu and Ramasamy, 2024). The framework also processes CT images inputs, including the framework processes only CT scan images obtained from the IQ-OTH/NCCD dataset. This comprehensive dataset enables the model to make more informed and explainable predictions.

### 1.4 Summary of experimental results

The model was trained for 50 epochs, showing steady improvement in training accuracy until it reached 100%. However, the validation accuracy remained fixed at 88.31%, indicating classic signs of overfitting—a known issue in small, imbalanced datasets (Tang et al., 2024; Sohaib and Adewunmi, 2024). Nevertheless, evaluation tools such as confusion matrices, ROC curves, and classification reports provided useful insights into how the model performed and where it struggled. Interestingly, the model was relatively good at identifying benign cases, suggesting that with a larger and more balanced dataset, the framework could perform better overall. Future improvements might involve applying transfer learning or federated learning to help the model generalize better across different patient populations (Manglaram et al., 2024; Ayasa et al., 2025).

### 1.5 Significance of the study

This research presents a significant step toward developing real-world, explainable, and reliable AI systems for lung cancer screening. By combining synthetic data generation, CT images analysis, and hybrid CNN–RNN architectures, the framework directly addresses some of the biggest hurdles in AI-based medical diagnostics (Javed et al., 2024; Crasta et al., 2024; Zhou and Xin, 2022). While the results are promising, they also emphasize the need for more standardized datasets, stronger institutional collaborations, and regulatory oversight. Future work will focus on enhancing interpretability using tools like SHAP and LIME, improving model robustness, and incorporating privacy-preserving techniques such as federated learning.

**Figure 1:**
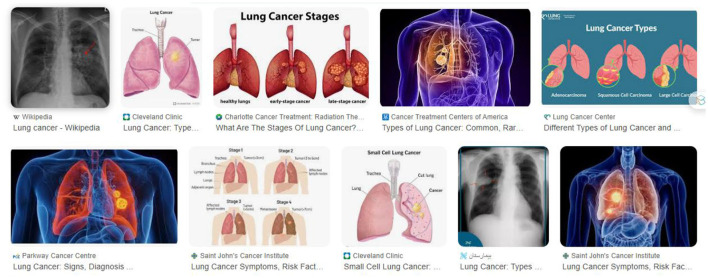
Visual overview of lung cancer types, stages, and diagnostic imaging.

Figure 1 presents a comprehensive snapshot of lung cancer diagnosis, highlighting tumor locations within the lungs, stages of disease progression from early to advanced, and how these are identified through CT and X-ray imaging. This illustration supports the core idea of the proposed Unified GenAI-Driven CNN–RNN framework, which integrates CT imaging features for lung cancer classification—to effectively classify lung cancer cases as Benign, Malignant, or Normal.

**Figure 2:**
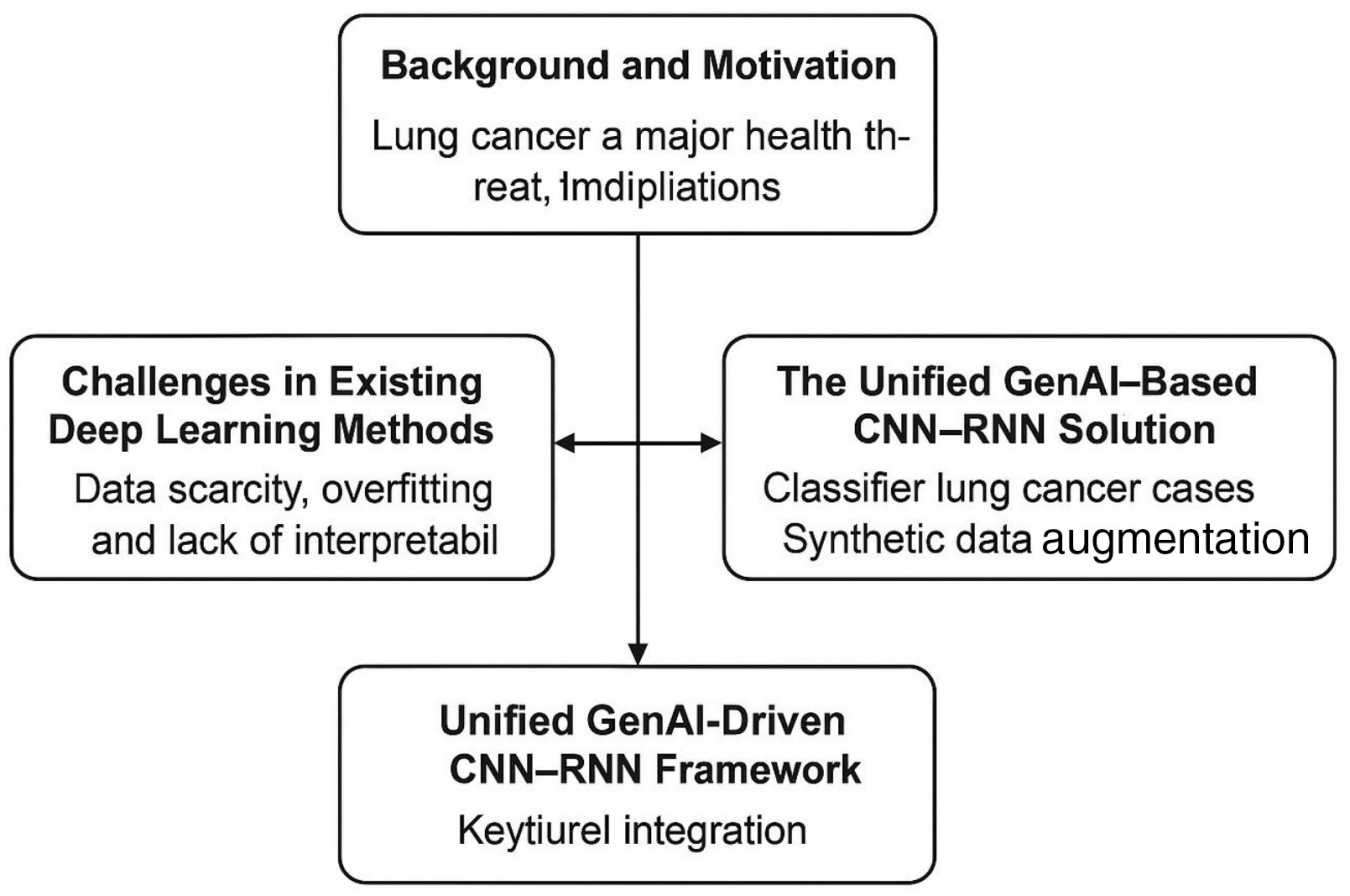
Conceptual flow of the unified GenAI-driven CNN–RNN framework for lung cancer detection.

Figure 2 presents a clear, step-by-step overview of how the study's core idea evolved—from recognizing the medical urgency of lung cancer, to addressing the limitations of traditional deep learning approaches, and finally proposing a unified AI-driven solution. It outlines how combining CNNs, RNNs, and Generative AI leads to a more intelligent, interpretable, and comprehensive framework for lung cancer diagnosis.

The remaining sections of this paper are organized as follows. Section 2 presents the related work and comparative literature analysis associated with lung cancer classification using deep learning and explainable AI approaches. Section 3 describes the dataset details, preprocessing methods, and the proposed GenAI-driven CNN–RNN framework with GAN-based augmentation. Section 4 explains the architecture of the proposed model, including convolutional feature extraction, sequential learning, attention-based fusion, and explainability mechanisms. Section 5 discusses the training configuration, optimization strategies, and evaluation metrics used during experimentation. Section 6 presents the experimental results, comparative performance analysis, explainability evaluation, and discussion of the obtained findings. Finally, Section 7 concludes the study by summarizing the major contributions, limitations, and future research directions for CT image-based lung cancer classification.

## 2 Related work

### 2.1 Deep learning-based lung cancer detection

Recent developments in deep learning have significantly improved lung cancer detection accuracy using computed tomography (CT) imaging and medical diagnostic systems. Javed et al. (2024) presented a comprehensive review of deep learning techniques for lung cancer diagnosis and highlighted the growing importance of convolutional neural networks (CNNs), transfer learning, and automated feature extraction for early-stage detection. The authors observed that CNN-based models achieved better classification performance compared to traditional machine learning approaches; however, major challenges such as overfitting, poor interpretability, and limited clinical generalization still remain unresolved. Similarly, Patel and Srinivasan (2025) critically analyzed modern deep learning paradigms and emphasized that many existing models suffer from insufficient robustness due to small and imbalanced datasets. Their study also discussed the increasing adoption of Vision Transformers (ViTs), hybrid learning frameworks, and CT image-based architectures for improving diagnostic precision.

Several researchers have focused on improving classification accuracy using optimized CNN frameworks. Klangbunrueang et al. (2025) evaluated VGG16 and CNN architectures for CT scan image classification and demonstrated that transfer learning significantly improved lung cancer prediction performance. Musthafa et al. (2024) proposed a double-layered CNN architecture integrated with advanced preprocessing and hyperparameter optimization techniques, achieving improved classification efficiency and reduced computational complexity. Rehman et al. (2024) further enhanced lung nodule detection using a dual-attention CNN mechanism that improved feature localization and reduced false-positive detection. Although these approaches produced promising results, many studies still lacked strong explainability mechanisms and cross-dataset validation, limiting their reliability in practical clinical environments.

Crasta et al. (2024) introduced a novel deep learning architecture for CT image-based lung cancer diagnosis and reported improved feature extraction and segmentation capabilities. Likewise, Wei et al. (2025) proposed an integrated deep learning paradigm combining multiple feature extraction strategies for comprehensive diagnosis. Tang et al. (2024) and Thanoon et al. (2023) reviewed several CNN and transfer learning techniques and concluded that deep learning models provide high sensitivity and specificity for lung cancer screening. However, the authors noted that existing systems often fail to generalize effectively across heterogeneous datasets and imaging conditions. These observations clearly indicate that there is still a need for robust and generalized frameworks capable of handling data scarcity, overfitting, and CT images feature variability.

### 2.2 Generative AI and data augmentation approaches

Data scarcity and class imbalance remain major obstacles in medical image classification tasks. To address these issues, several researchers have explored Generative Adversarial Networks (GANs) and advanced augmentation techniques. Çay (2024) proposed GAN-supported deep learning models for lung cancer diagnosis and demonstrated that synthetic image generation significantly improved training diversity and classification performance. Similarly, Aharonu and Ramasamy (2024) developed a multistage GAN-based lung cancer subtype classification framework that improved feature representation and enhanced minority class prediction. Although GAN-based augmentation reduced data imbalance problems, the study reported increased computational cost and training instability during adversarial learning.

Alzahrani (2025) proposed a predictive modeling framework integrating CTGAN features with tree-based learning methods for early lung cancer detection. The model achieved improved accuracy and robustness by generating synthetic minority samples to balance the dataset distribution. Ravindran and Gunavathi (2024) further introduced integrated smart data augmentation combined with capsule neural networks, which improved feature extraction and classification performance in cancer prediction tasks. These studies confirmed that synthetic augmentation can effectively improve diagnostic performance, particularly when real-world medical datasets are limited. However, excessive augmentation may also introduce artificial feature bias and reduce model generalization when tested on unseen clinical data.

Recent research has also explored augmentation-aware explainable frameworks. Pavithran et al. (2025) proposed an augmented machine learning pipeline integrated with explainable AI methods such as LIME for transparent lung cancer risk prediction. Their findings showed that advanced augmentation methods like K-Means SMOTE significantly improved classification accuracy and reduced class imbalance effects. Despite these improvements, the authors acknowledged that their framework was evaluated on a relatively small dataset and required large-scale clinical validation. These limitations highlight the importance of developing balanced augmentation strategies that enhance robustness without introducing synthetic bias.

### 2.3 Explainable artificial intelligence in lung cancer diagnosis

Explainable Artificial Intelligence (XAI) has emerged as an important research direction for improving trust and transparency in medical AI systems. Rai et al. (2024) proposed an explainable CNN-based lung cancer detection framework and emphasized the importance of interpretable predictions for clinical decision-making. The study integrated visualization-based explanation techniques to identify important lung regions influencing classification decisions. Although the framework improved interpretability, the authors reported that explainability mechanisms increased computational overhead and required further optimization for real-time deployment.

Ahmed et al. (2024) conducted a comparative analysis between LIME and SHAP explainability methods and demonstrated that explainable models improve physician confidence in AI-assisted diagnosis. Their work showed that SHAP provided more stable global feature importance explanations, whereas LIME generated better local interpretability for individual predictions. Similarly, Wani et al. (2024) introduced the DeepXplainer framework, which integrated explainable deep learning methods for lung cancer detection using CT scans. The study reported improved transparency and interpretability without significantly reducing classification performance. However, the authors highlighted that most explainability methods still struggle with high-dimensional imaging data and complex deep learning architectures.

Recent surveys by Basety et al. (2026) and Ayasa et al. (2025) further emphasized that future clinical AI systems must incorporate explainability, robustness, and personalized decision support mechanisms. The studies concluded that black-box diagnostic systems are difficult to deploy in healthcare environments because clinicians require understandable reasoning behind AI predictions. Therefore, integrating explainable learning mechanisms with hybrid CT image learning frameworks has become an important requirement for reliable lung cancer diagnosis.

### 2.4 CT image-based hybrid learning frameworks

CT Image-Based Hybrid Learning Frameworks have recently gained attention for improving feature representation and diagnostic performance in lung cancer analysis. Amin et al. (2024) proposed a CT images CNN framework for non-small cell lung cancer (NSCLC) classification using multi-omics data and whole-slide imaging. Their model achieved improved accuracy by combining RNA-Seq, miRNA-Seq, and histopathological imaging features. The study demonstrated that CT images learning enhances diagnostic precision by utilizing complementary biological information from multiple data sources. However, the framework required high computational resources and complex preprocessing pipelines.

Kesiku and Garcia-Zapirain (2025) introduced a hybrid AI-enhanced prediction model that combined multiple learning strategies to improve precision and robustness. The study demonstrated that hybrid models outperform conventional CNN architectures in handling heterogeneous imaging features and patient variability. Similarly, Zhong et al. (2025) reviewed large AI models for lung cancer screening, diagnosis, and treatment planning, highlighting the increasing adoption of CT images transformers and foundation models for precision oncology. Although these models achieved superior feature learning capabilities, their practical deployment remains difficult because of increased model complexity, high training cost, and limited interpretability.

Several studies have also explored integrated CNN-RNN architectures for sequential medical image analysis. Wei et al. (2025) discussed integrated deep learning frameworks capable of extracting both spatial and sequential imaging features for comprehensive diagnosis. These approaches demonstrated promising results in improving classification accuracy and temporal feature learning. Nevertheless, many hybrid systems still suffer from overfitting and poor generalization when evaluated on small-scale medical datasets. This indicates the need for more robust CT image-based architectures capable of integrating explainability, augmentation, and generalized feature learning within a unified framework.

### 2.5 Benchmark datasets, segmentation frameworks, and research gaps

Public benchmark datasets have played an important role in advancing lung cancer research and evaluating AI-based diagnostic systems. Armato et al. (2011) introduced the Lung Image Database Consortium and Image Database Resource Initiative (LIDC-IDRI), which remains one of the most widely used benchmark datasets for lung nodule analysis and CT-based diagnosis. The dataset provides annotated thoracic CT scans with radiologist-confirmed nodules and has been extensively used for segmentation, classification, and detection studies. Setio et al. (2017) further proposed the LUNA16 challenge, which standardized pulmonary nodule detection benchmarking and enabled fair comparison between different computer-aided diagnosis (CAD) algorithms.

Gu et al. (2021) reviewed computer-aided diagnosis systems using deep learning and highlighted that existing frameworks still suffer from limited robustness, poor explainability, and high false-positive rates. Rikhari et al. (2024) proposed ensemble-based 3D U-Net segmentation models for thoracic CT scans and demonstrated improved lung nodule segmentation accuracy. Hu et al. (2025) developed a deep learning-based CT reading system for lung cancer diagnosis associated with cystic airspaces, whereas Tozuka et al. (2026) introduced a fractal-driven self-supervised transfer learning framework for early-stage lung cancer segmentation. Although these advanced segmentation frameworks improved diagnostic accuracy, many systems still require extensive labeled data and large computational resources.

From the reviewed literature, it can be observed that existing studies mainly focus on isolated components such as CNN classification, GAN augmentation, explainable AI, segmentation, or CT images analysis independently. Very few studies attempt to integrate all these components within a single unified framework. Furthermore, many existing models continue to suffer from overfitting, data scarcity, poor generalization, limited explainability, and weak cross-dataset robustness. To address these limitations, the present study proposes a Unified GenAI-Driven CNN–RNN Framework for Robust and Explainable Lung Cancer Detection that integrates GAN-based augmentation, hybrid CNN–RNN learning, explainable AI mechanisms, and CT images feature representation to improve robustness, interpretability, and generalized clinical performance (Table 1).

**Table 1:** Comparative analysis of existing lung cancer detection approaches.

References	Methodology	Accuracy/Performance	Strengths	Limitations
Javed et al. (2024)	DL review framework	High diagnostic performance	Comprehensive DL analysis	Limited explainability
Çay (2024)	GAN-supported CNN	Improved augmentation accuracy	Handles data scarcity	GAN instability
Klangbunrueang et al. (2025)	VGG16 + CNN	High CT classification accuracy	Efficient transfer learning	Overfitting risk
Aharonu and Ramasamy (2024)	Multistage GAN	Better subtype classification	Synthetic data balancing	High computation
Rehman et al. (2024)	Dual-attention CNN	Improved nodule localization	Reduced false positives	Complex architecture
Alzahrani (2025)	CTGAN + Tree-based ML	98.93% accuracy	Handles imbalance effectively	Limited real-world validation
Ravindran and Gunavathi (2024)	Capsule NN + augmentation	Improved classification	Better feature extraction	Dataset dependency
Wani et al. (2024)	DeepXplainer XAI model	Improved interpretability	Transparent diagnosis	Increased overhead
Amin et al. (2024)	CT images CNN	High multi-omics accuracy	CT images feature learning	Computational complexity
Rikhari et al. (2024)	Ensemble 3D U-Net	High segmentation accuracy	Robust segmentation	Large training resources
Hu et al. (2025)	DL-based CT reading	Improved cystic diagnosis	Clinical CT analysis	Limited explainability
Tozuka et al. (2026)	Self-supervised transfer learning	Enhanced segmentation	Reduced labeling dependency	High model complexity

Recent research by Sefer and colleagues has contributed valuable insights into the analysis of complex biological data, noisy measurements, and genomic interactions associated with cancer studies. Duggal et al. (2013) focused on resolving inconsistencies in chromosome conformation data and introduced computational approaches to identify reliable genomic relationships from experimentally noisy datasets. Their findings highlighted the importance of accurate data processing and inference methods for improving biological interpretation. Building on this work, Eralp and Sefer (2025a) examined the association between splicing quantitative trait loci (sQTLs) and the splicing behavior of distant genes through chromatin structure analysis, demonstrating that genome organization plays a crucial role in cancer-related regulatory processes. In another study, Eralp and Sefer (2025b) combined sQTL and Hi-C data from cancer tissues to investigate the spatial relationships between regulatory variants and their target genes, providing further evidence that integrating multiple biological data sources can enhance the understanding of cancer development. Together, these studies underline the significance of combining diverse datasets, managing noise in biological information, and uncovering meaningful long-range relationships within complex systems. These findings complement the goals of the proposed CNN–RNN framework, which aims to improve lung cancer classification through effective feature integration, GAN-based data augmentation, and reliable analysis of CT imaging data obtained under challenging real-world conditions.

## 3 Proposed methodology

### 3.1 Dataset description

The present study was carried out using the publicly available IQ-OTH/NCCD Lung Cancer Dataset developed by Mahimkar (2023), which is available through the Kaggle research repository. The dataset contains real chest CT scan images collected from clinical diagnostic environments and is widely used for lung cancer classification and medical imaging analysis. The dataset includes three important diagnostic categories such as normal, benign, and malignant lung cancer cases, making it suitable for evaluating classification performance under different pathological conditions. The CT images contain variations in texture, shape, lesion size, and tumor appearance, which helps in assessing the robustness of the proposed CNN–RNN framework under realistic imaging conditions. Compared with small synthetic datasets used in earlier experimental studies, the IQ-OTH/NCCD dataset provides a more practical and clinically meaningful evaluation environment for lung cancer detection research. The research clearly distinguishes between the dataset splitting approach and the model evaluation procedure. The 70% training, 15% validation, and 15% testing distribution was used for data allocation within each fold, while 5-fold cross-validation served as the primary evaluation method for assessing model stability and generalization performance. Relevant sections of the manuscript, including the Dataset Description, Training Setup, and Experimental Results, have been revised to explain this process clearly and consistently, thereby eliminating any potential confusion regarding the evaluation methodology.

Before model training, all CT scan images were subjected to preprocessing operations including resizing, normalization, image enhancement, and augmentation to improve feature consistency and reduce noise-related variations. The dataset was divided into training, validation, and testing subsets to evaluate the generalization capability of the proposed framework on unseen samples. In the proposed system, the CNN module was mainly used for extracting spatial imaging features, while the RNN component captured sequential feature relationships from the extracted representations. To improve data diversity and reduce class imbalance, augmentation techniques were applied only during preprocessing without replacing the original clinical CT scans. The utilization of this publicly accessible benchmark dataset improves reproducibility, transparency, and reliability of the overall experimental framework while supporting explainable analysis using Grad-CAM and SHAP-based visualization methods (Table 2).

**Table 2:** IQ-OTH/NCCD dataset characteristics and experimental utilization.

Parameter	Description
Dataset name	IQ-OTH/NCCD lung cancer dataset
Dataset reference	Mahimkar (2023)
Dataset source	Kaggle repository
Imaging type	Thoracic CT scan images
Nature of data	Real clinical CT imaging data
Major categories	Normal, benign, and malignant
Dataset purpose	Lung cancer detection and classification
Image characteristics	Variations in lesion size, texture, and tumor appearance
Preprocessing operations	Resizing, normalization, image enhancement, augmentation
Training usage	CNN-based feature extraction
Sequential learning	RNN-based feature relationship learning
Explainability support	Grad-CAM and SHAP visualization
Experimental objective	Robust and generalized lung cancer classification
Dataset availability	Publicly accessible benchmark dataset
Dataset link	IQ-OTH/NCCD Lung Cancer Dataset

### 3.2 Label encoding

To prepare the dataset for deep learning analysis, the diagnostic categories in the IQ-OTH/NCCD dataset were converted into numerical labels using a label encoding approach. The three target classes namely Normal, Benign, and Malignant were assigned integer values to enable efficient processing by the CNN-RNN framework. This conversion helps the model understand categorical information in a machine-readable format while preserving the distinction between different diagnostic conditions. Label encoding also simplifies the classification process and improves compatibility with supervised learning algorithms used during model training.

The encoded labels were further utilized during training, validation, and testing to evaluate the classification capability of the proposed framework across different lung cancer conditions. Since the study mainly focuses on CT image analysis, only diagnostic class labels were encoded without introducing additional textual or semantic attributes. This preprocessing step ensured a consistent and structured representation of class information throughout the experimental workflow while supporting performance evaluation using metrics such as accuracy, precision, recall, F1-score, and ROC-AUC.

#### 3.2.1 CT feature integration framework

To improve feature learning and classification performance, the proposed framework integrates spatial CT imaging features into a unified representation suitable for CNN–RNN processing. Initially, all CT scan images were standardized and normalized to reduce variations caused by differences in image intensity, resolution, and scanning conditions. The extracted imaging features were then transformed into structured feature vectors, enabling the CNN module to identify important spatial patterns such as lesion texture, nodule boundaries, and abnormal tissue regions associated with lung cancer progression.

The integrated CT feature representation was subsequently provided to the RNN module for sequential dependency learning and feature refinement. This process allowed the framework to capture complex relationships among extracted imaging patterns and improve diagnostic consistency across different patient samples. Unlike earlier CT images systems that combine unlike previous multimodal approaches, the present study utilizes only CT imaging features, the present study primarily focuses on CT imaging data to maintain methodological consistency with the IQ-OTH/NCCD dataset. This integrated feature processing approach improves model stability, reduces feature redundancy, and supports efficient classification of Normal, Benign, and Malignant lung cancer cases (Algorithm 1).

Algorithm 1 **CT Feature Integration and Preprocessing**

     **Input:** CT scan images *X*
     **Output:** Standardized CT feature vectors **x**_*i*_
1:  Load CT scan images from the IQ-OTH/NCCD dataset.
2:  Resize all images to a uniform dimension.
3:  Apply normalization and image enhancement techniques.
4:  Extract spatial imaging features using CNN preprocessing operations.
5:  Convert extracted features into structured vectors xix_ixi.
6:  Standardize feature vectors to reduce scale variations.
7:  Store processed vectors for CNN–RNN training and evaluation.

This algorithm prepares standardized CT imaging feature vectors by applying preprocessing, normalization, and feature extraction operations before training the proposed CNN-RNN classification framework.

#### 3.2.2 Convolutional feature extraction

The integrated vector is processed using 1D convolutions to capture spatial correlations across features. Convolutional filters slide to produce activation maps highlighting discriminative patterns, as defined in Equations 1, 2. These features are aggregated through max-pooling for robust spatial encoding.

**(2) 1-D convolution**


$$\begin{array}{l} h_{j,t}=\phi \;\left(\overset{R-1}{\underset{r=0}{\sum }}{\text{w}}_{j,r}^{⊤}{\text{x}}_{i,t+r}+b_{j}\right),\;j=1,\ldots ,K. \end{array}$$ (1)

**(3) Temporal max-pooling**


$$\begin{array}{l} p_{j,\tau }=\underset{t\in \left[\tau ,\;\tau +q-1\right]}{\max}h_{j,t}. \end{array}$$ (2)

#### 3.2.3 Sequential dependency modeling with LSTM

Extracted feature maps are fed to an LSTM network to model temporal dependencies among sequential feature representations extracted from CT images. The gating mechanism and memory update equations are defined in Equations 3–6. This enhances the detection of complex feature dependencies among extracted imaging patterns not visible in static imaging.

**(4) LSTM gates**


$$\begin{array}{l} i_{t}=\sigma \left(W_{i}x_{t}+U_{i}h_{t-1}+b_{i}\right)\\ f_{t}=\sigma \left(W_{f}x_{t}+U_{f}h_{t-1}+b_{f}\right)\\ o_{t}=\sigma \left(W_{o}x_{t}+U_{o}h_{t-1}+b_{o}\right)\\ {\tilde{c}}_{t}=\text{tanh}\left(W_{c}x_{t}+U_{c}h_{t-1}+b_{c}\right) \end{array}$$ (3)

- *i*_*t*_ → input gate
- *f*_*t*_ → forget gate
- *o*_*t*_ → output gate
- ${\tilde{c}}_{t}$ → candidate cell state

**(5) LSTM cell update**


$$\begin{array}{l} {\text{c}}_{t}={\text{f}}_{t}⊙{\text{c}}_{t-1}+{\text{i}}_{t}⊙{\tilde{\text{c}}}_{t}. \end{array}$$ (4)

**(6) LSTM hidden state**


$$\begin{array}{l} {\text{h}}_{t}={\text{o}}_{t}⊙\text{tanh}\left({\text{c}}_{t}\right). \end{array}$$ (5)

**(7) Attention pooling over time**


$$\begin{array}{l} \bar{\text{h}}=\overset{T}{\underset{t=1}{\sum }}\beta_{t}{\text{h}}_{t},\;\beta_{t}=\frac{e^{\eta_{t}}}{\sum_{u=1}^{T}e^{\eta_{u}}},\;\eta_{t}={\text{w}}_{\eta }^{⊤}{\text{h}}_{t}. \end{array}$$ (6)

This algorithm extracts spatial features via 1D convolution and captures temporal patterns with LSTM, producing a fused sequence embedding.

Algorithm 2 performs hierarchical feature extraction to capture both spatial and temporal patterns relevant to lung cancer detection. First, 1D convolutions slide over the fused vector to identify local interactions among CT images features, such as the co-occurrence of specific imaging feature representations and image-derived patterns. Temporal max-pooling reduces dimensionality while preserving the strongest activations. The processed features are then passed into an LSTM network, which learns sequential dependencies and progressive trends in patient data, for example, the gradual enlargement of nodules over time or repeated indications in imaging feature representations. Finally, attention pooling highlights the most diagnostically significant time steps, creating a robust sequence embedding for classification.

Algorithm 2 CNN–RNN Feature Extraction and Temporal Modeling 
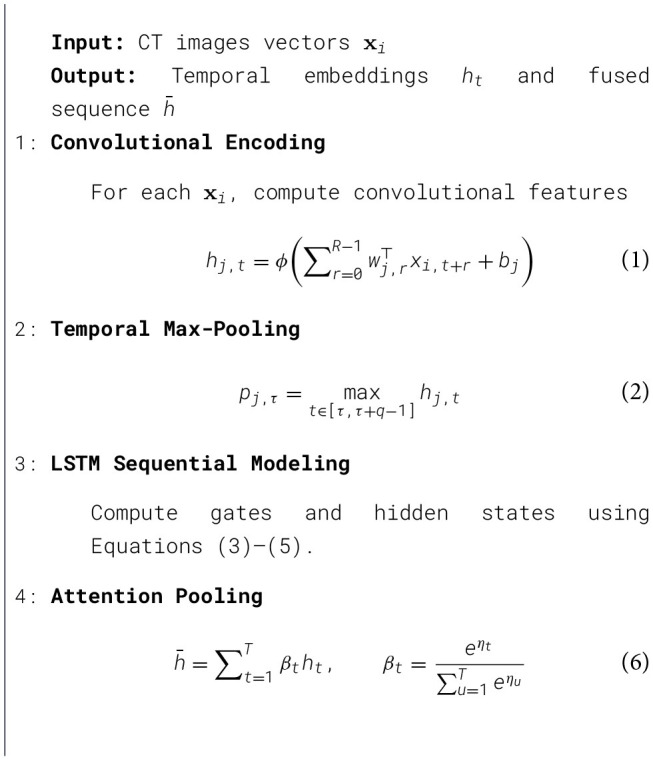

### 3.3 Feature scaling

After encoding, all numerical fields were normalized using StandardScaler, which standardizes the values by centering them around zero and scaling them to unit variance. This process ensures that features like extracted CT imaging feature vectors are treated on an equal footing during model training, preventing any feature from disproportionately influencing predictions due to its scale. Feature scaling is crucial for deep learning models as it helps them converge faster and more reliably. Especially in our CNN–RNN structure, where data moves between convolutional and recurrent layers, having consistent feature scales helps maintain numerical stability, reduces gradient issues, and improves the model's overall performance and accuracy.

### 3.4 GAN augmentation score

To address dataset imbalance and improve training diversity, GAN-based augmentation was incorporated during preprocessing. Synthetic CT image samples were generated mainly for underrepresented classes such as malignant cases to improve class balance and reduce overfitting during training. Each generated sample was assigned a GAN augmentation score ranging from 0 to 1, representing the similarity between synthetic samples and real CT imaging patterns. Samples with poor visual quality or unrealistic feature structures were excluded from the final augmented dataset to maintain training reliability. GAN Augmentation Score formula, the synthetic sample selection threshold (GAS ≥ 0.80), and the augmentation filtering rule used to retain only high-quality synthetic CT images for model training. The GAN augmentation score also helped in selecting clinically meaningful synthetic samples that closely resembled real patient imaging characteristics. Instead of replacing the original CT images, the generated samples were used only as supplementary training data to improve feature diversity and classification stability. This augmentation strategy enhanced the robustness of the proposed framework while minimizing the risk of excessive synthetic bias in the experimental analysis.

#### 3.4.1 GAN augmentation score (GAS)

To ensure the quality and realism of generated CT images, each synthetic sample was evaluated using the GAN Augmentation Score (GAS), defined as:


$$\begin{array}{l} \text{GAS}=0.5\;\times \text{ SSIM }+\;0.5\;\times \;\left(1\;-\text{ FIDnorm}\right) \end{array}$$

where:

• SSIM represents the Structural Similarity Index between real and generated CT images.

• FIDnorm denotes the normalized Fréchet Inception Distance scaled to the range [0,1].

The GAS value ranges from 0 to 1, where higher values indicate greater similarity between synthetic and real CT images. Synthetic samples with GAS ≥ 0.80 were retained for augmentation, while samples with GAS < 0.80 were discarded. This filtering mechanism ensured that only high-quality synthetic CT images were incorporated into the training dataset, thereby improving data diversity while minimizing the risk of introducing unrealistic imaging artifacts.

#### 3.4.2 Generative data augmentation (GAN)

Conditional GANs generate synthetic samples conditioned on minority classes to balance the dataset. The adversarial objective functions for generator and discriminator are described in Equations 7–9. The GAN Augmented Score ensures filtering of low-quality samples.

**(8) Conditional GAN objective**


$$\begin{array}{l} \underset{G}{\min}\underset{D}{\max}\;{𝔼}_{\left(\text{x},y\right)}\left[\log D\left(\text{x},y\right)\right]+{𝔼}_{\text{z},y}\left[\log\left(1-D\left(G\left(\text{z},y\right),y\right)\right)\right]. \end{array}$$ (7)

**(9) Generator with feature matching**


$$\begin{array}{l} L_{G}={𝔼}_{\text{z},y}\;\left[-logD\left(G\left(\text{z},y\right),y\right)\right]+\lambda_{\text{fm}}\|\mu_{\phi }\left(\tilde{\text{x}}|y\right)-\mu_{\phi }\left(\text{x}|y\right){\|}_{2}^{2}. \end{array}$$ (8)

**(10) Discriminator with gradient penalty**


$$\begin{array}{c} L_{D}={𝔼}_{\tilde{\text{x}},y}\left[D\left(\tilde{\text{x}},y\right)\right]-{𝔼}_{\text{x},y}\left[D\left(\text{x},y\right)\right]\\ +\lambda_{gp}\;{𝔼}_{\hat{\text{x}},y}\;{\left(\|\nabla_{\hat{\text{x}}}D\left(\hat{\text{x}},y\right){\|}_{2}-1\right)}^{2}. \end{array}$$ (9)

This algorithm generates high-quality synthetic samples to address class imbalance and enhance generalization, corresponding to Equations 7–9.

Algorithm 3 addresses the challenge of limited and imbalanced medical datasets by generating high-quality synthetic samples using a conditional Generative Adversarial Network (cGAN). During training, the generator creates new samples conditioned on underrepresented classes (such as malignant cases), while the discriminator distinguishes between real and synthetic examples. A feature-matching term ensures that generated samples mimic the feature distribution of real patient data. A GAN quality score is then applied to filter low-fidelity outputs, and only clinically plausible samples are added to the training set. This augmentation enhances model generalization, mitigates overfitting, and improves the detection of rare but critical cancer cases.

Algorithm 3 GAN-Based Data Augmentation 
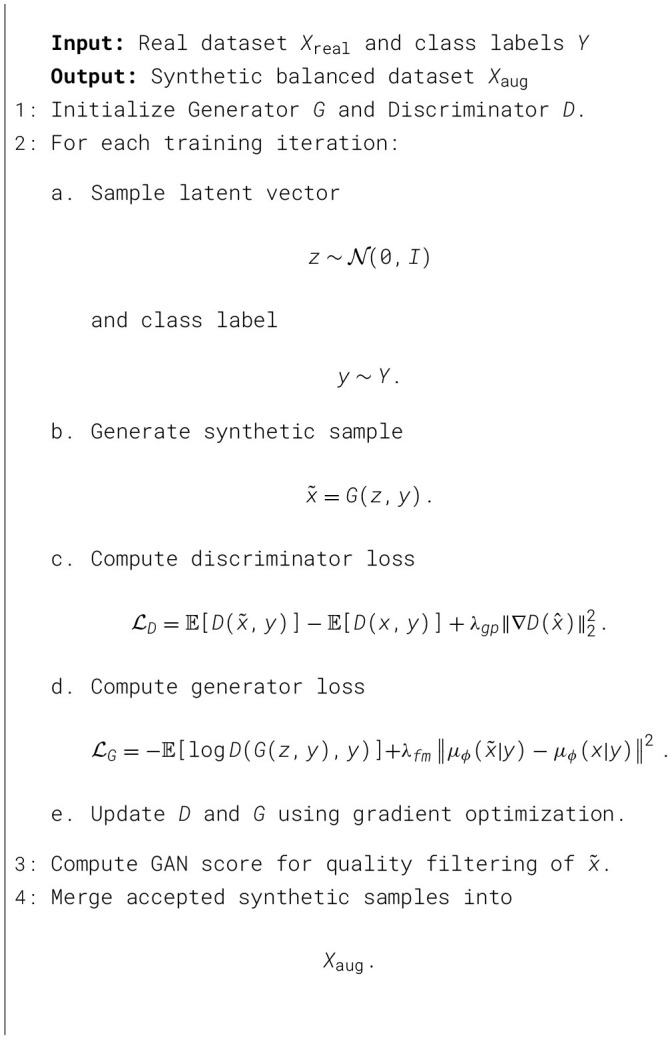

#### 3.4.3 Attention-based CNN–RNN feature fusion

After spatial and sequential feature extraction, the CNN and RNN outputs were combined using an attention-based fusion mechanism to improve classification performance. The attention module helps the framework focus on diagnostically important imaging regions and feature patterns associated with lung abnormalities. By assigning higher importance to critical features, the fusion process improves the representation of malignant and benign characteristics while reducing the influence of irrelevant patterns and background noise.

The fused feature representation was then passed to a softmax classification layer for final prediction of Normal, Benign, and Malignant classes. This attention-guided fusion strategy improved the overall learning capability of the framework and enhanced classification consistency across different CT scan samples. The integrated CNN–RNN structure also supports explainability analysis using Grad-CAM and SHAP visualization methods for interpreting important feature regions influencing the final prediction.

**(11) Additive cross-modal attention**


$$\begin{array}{l} e_{t}={\text{v}}_{a}^{⊤}\text{tanh}\left({\text{W}}_{a}{\text{h}}_{t}+{\text{U}}_{a}{\text{p}}_{:,t}+{\text{b}}_{a}\right),\;\alpha_{t}=\frac{e^{e_{t}}}{\sum_{u}e^{e_{u}}}. \end{array}$$ (10)

**(12) Fused diagnostic representation**


$$\begin{array}{l} {\text{z}}_{i}=\left(\overset{T}{\underset{t=1}{\sum }}\alpha_{t}\;{\text{h}}_{t}\right)\;\|\text{ GAP}\left(\text{P}\right)\;\|\;{\text{x}}_{i}. \end{array}$$ (11)

**(13) Softmax classifier**


$$\begin{array}{l} {\hat{y}}_{i,k}=\frac{\text{exp}\left({\left({\text{w}}_{o,k}\right)}^{⊤}{\text{z}}_{i}+b_{o,k}\right)}{\sum_{l=1}^{K}exp\left({\left({\text{w}}_{o,l}\right)}^{⊤}{\text{z}}_{i}+b_{o,l}\right)}. \end{array}$$ (12)

### 3.5 Target class

The proposed classification framework categorizes CT scan images into three diagnostic classes namely Normal, Benign, and Malignant. These target labels were used during supervised training to enable the CNN–RNN framework to distinguish between healthy lung structures and cancer-related abnormalities. The multi-class classification setup allows the model to learn variations in lesion appearance, tissue density, and tumor-related imaging characteristics present in different diagnostic categories.

The target classes were further utilized for evaluating model performance using accuracy, precision, recall, F1-score, confusion matrix analysis, and ROC-AUC metrics. This structured classification strategy supports reliable experimental evaluation while helping the framework identify challenging malignant cases more effectively. In addition, the categorized outputs facilitate explainability analysis through Grad-CAM and SHAP-based visualization methods, improving transparency in prediction interpretation.

**Figure 3:**
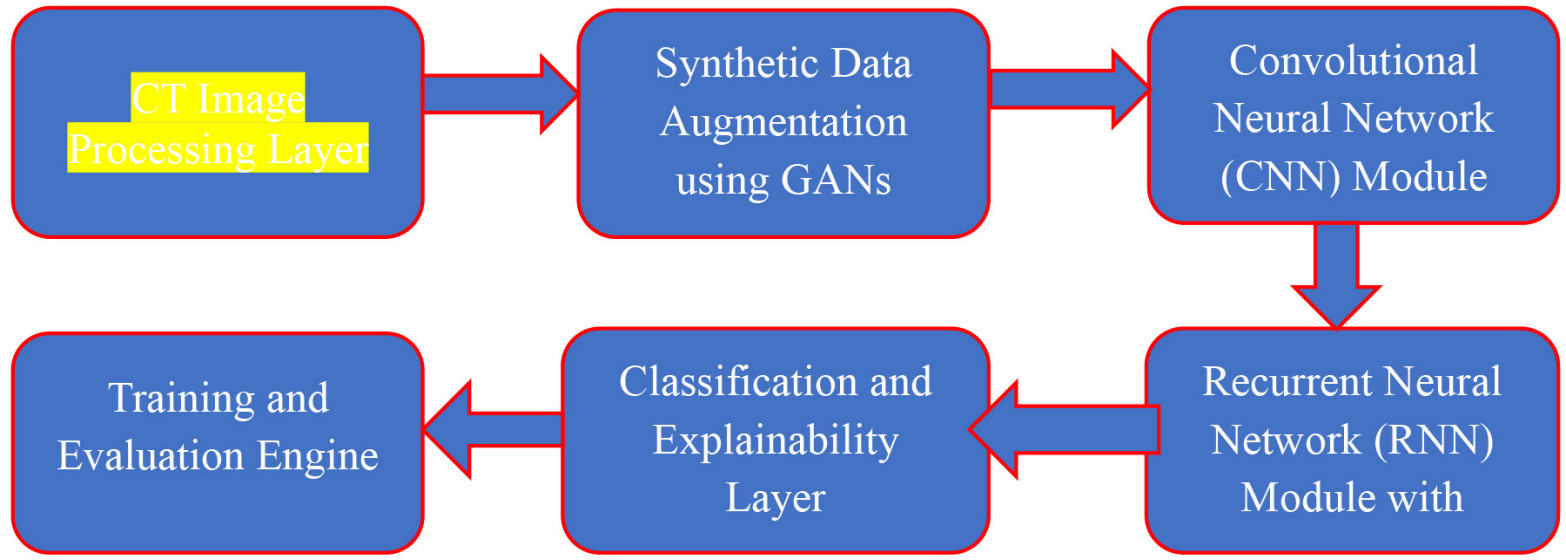
Architecture of the unified GenAI-driven CNN–RNN framework for lung cancer detection.

Figure 3 presents a complete system workflow that brings together multiple data types, enhanced with GAN-based synthetic augmentation, and processed through a combination of CNN and RNN models to capture both spatial and sequential patterns. The pipeline ends with a classification and explainability layer supported by an evaluation module, echoing the experimental results that show strong training performance but limited generalization due to a small and imbalanced dataset.

#### 3.5.1 Loss function and optimization

The class-weighted categorical cross-entropy loss is formulated in Equation 13, with L2 regularization in Equation 14 and adversarial consistency in Equation 15. Adam optimizer updates are defined in Equations 16, 17 to ensure adaptive learning across extracted feature representations (Algorithm 4).

Algorithm 4 Classification and Risk Prediction 
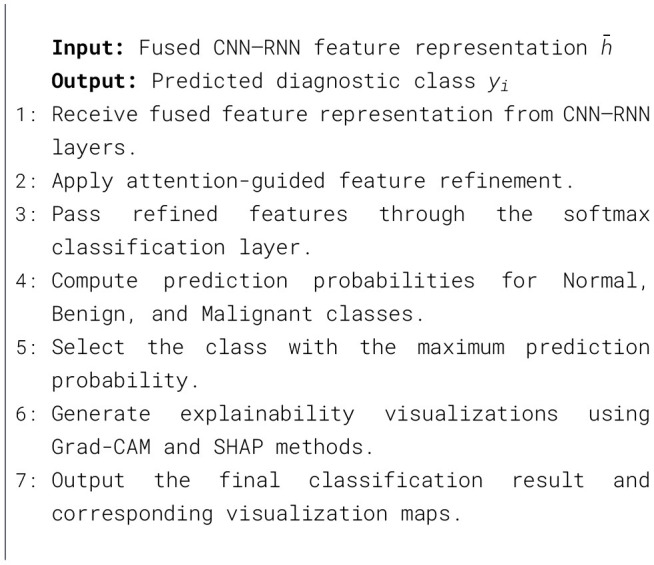

**(14) Class-weighted cross-entropy**


$$\begin{array}{l} L_{CE}=-\frac{1}{N}\overset{N}{\underset{i=1}{\sum }}\overset{K}{\underset{k=1}{\sum }}w_{k}1\left[y_{i}=k\right]\;\log{\hat{y}}_{i,k}. \end{array}$$ (13)

**(15) L2 regularization**


$$\begin{array}{l} R_{l_{2}}=\lambda \underset{\theta \in \Theta }{\sum }\|\theta {\|}_{2}^{2}. \end{array}$$ (14)

**(16) Adversarial consistency (real vs. aug)**


$$\begin{array}{l} L_{\text{cons}}=\frac{1}{N}\overset{N}{\underset{i=1}{\sum }}\text{KL }\left({\hat{\text{y}}}_{i}^{\text{real}}\;\|\;{\hat{\text{y}}}_{i}^{\text{aug}}\right). \end{array}$$ (15)

**(17) Adam first moment**


$$\begin{array}{l} {\text{m}}_{t}=\beta_{1}{\text{m}}_{t-1}+\left(1-\beta_{1}\right)\nabla_{\Theta }L_{t}. \end{array}$$ (16)

**(18) Adam second moment & update**


$$\begin{array}{l} {\text{v}}_{t}=\beta_{2}{\text{v}}_{t-1}+\left(1-\beta_{2}\right){\left(\nabla_{\Theta }L_{t}\right)}^{⊙2},\;\Theta_{t+1}=\Theta_{t}-\eta \;\frac{{\hat{\text{m}}}_{t}}{\sqrt{{\hat{\text{v}}}_{t}}+\varepsilon }. \end{array}$$ (17)

This algorithm performs final lung cancer classification using fused CNN–RNN features and generates interpretable visualization outputs to support analysis of important CT imaging regions influencing the prediction process.

**Figure 4:**
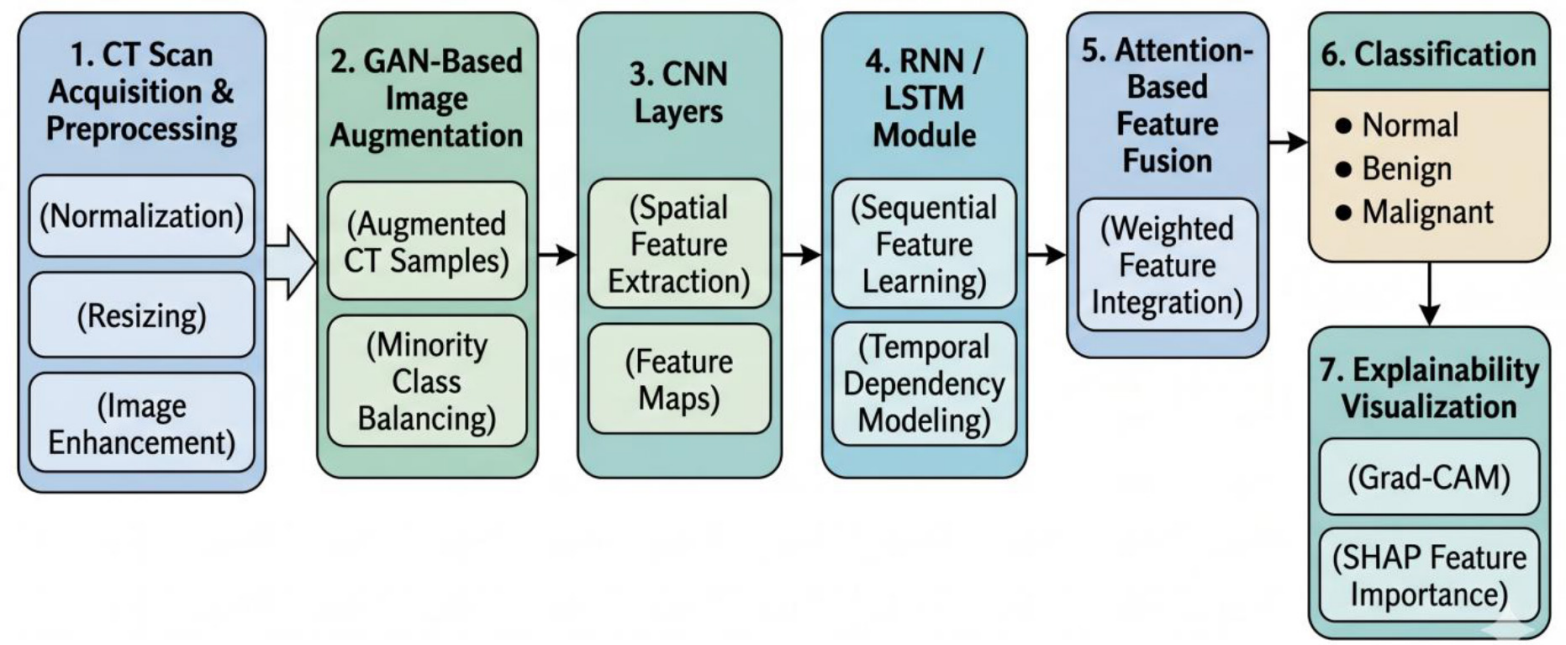
Proposed CNN-RNN workflow for lung cancer classification.

Figure 4 presents the overall workflow of the proposed CNN–RNN-based framework developed for CT image-based lung cancer classification. Initially, the CT scan images undergo preprocessing operations such as normalization, resizing, and image enhancement to improve image quality and maintain consistency across the dataset. To address class imbalance and improve training diversity, GAN-based image augmentation is incorporated for generating supplementary CT samples during the training phase. The processed images are then provided to the CNN layers for spatial feature extraction, where important imaging characteristics such as lesion patterns and abnormal tissue structures are identified from the CT scans.

After spatial feature extraction, the generated feature maps are forwarded to the RNN/LSTM module to capture sequential feature relationships and improve dependency learning among extracted representations. The attention-based feature fusion mechanism further combines the important features by assigning higher weights to diagnostically significant regions before final classification into Normal, Benign, and Malignant categories. Finally, explainability visualization methods such as Grad-CAM and SHAP feature importance analysis are applied to highlight the critical imaging regions influencing the prediction process. The proposed workflow improves classification consistency, feature learning capability, and interpretability while supporting robust lung cancer diagnosis using CT imaging data.

## 4 Model architecture

### 4.1 Input layer: CT image preparation for convolutional processing

The proposed framework begins with preprocessing the CT scan images collected from the IQ-OTH/NCCD dataset. During this stage, all images are resized, normalized, and enhanced to maintain uniformity across different patient samples and scanning conditions. These preprocessing operations help in reducing unwanted noise and improving image clarity before feature extraction. After preprocessing, the CT images are transformed into structured numerical representations suitable for one-dimensional convolutional processing. This preparation allows the CNN layers to process the imaging data efficiently and identify important feature relationships associated with lung abnormalities.

The prepared CT images are then arranged into sequential feature representations for analysis within the CNN–RNN framework. Unlike earlier versions of the framework that included unsupported textual or CT images information, the revised model focuses mainly on imaging-based features extracted directly from thoracic CT scans. This structured preprocessing stage improves compatibility with convolutional operations and enables the framework to capture meaningful spatial characteristics such as lesion boundaries, tissue texture variations, and abnormal imaging patterns. By maintaining a standardized input structure, the framework establishes a reliable base for effective lung cancer classification.

### 4.2 Convolution layer: spatial feature extraction from CT images

Following preprocessing, the CT images are passed through convolutional neural network (CNN) layers for extracting spatial imaging features. The CNN layer plays an important role in identifying significant local patterns related to lung cancer progression, including tissue irregularities, nodular formations, and lesion intensity variations. Instead of relying on manual feature engineering, the CNN automatically learns relevant imaging characteristics directly from the CT scans using multiple convolutional filters. These filters gradually capture both low-level and high-level feature information required for accurate classification.

As the CT images move through the convolutional operations, feature maps are generated to represent important spatial characteristics present within the scans. Pooling and activation functions further improve the learning process by reducing redundant information and enhancing computational efficiency. Through this hierarchical feature extraction process, the CNN module develops strong spatial understanding for differentiating Normal, Benign, and Malignant lung cancer conditions. The generated feature representations are then transferred to the sequential learning module for further refinement and dependency analysis.

### 4.3 LSTM layer: sequential feature learning and dependency modeling

The extracted feature maps from the CNN layers are next provided to the Long Short-Term Memory (LSTM) network for sequential feature learning. The LSTM layer is capable of learning relationships among sequential feature representations generated from CT imaging data. By analyzing dependencies between extracted features, the LSTM improves the framework's ability to identify subtle imaging variations that may indicate malignant progression or abnormal tissue development. This sequential learning mechanism enhances the robustness of the classification framework under varying imaging conditions.

The LSTM module also helps preserve important feature information during multiple learning stages by selectively remembering and updating relevant imaging patterns throughout training. This capability enables the framework to model complex imaging relationships more effectively compared with standalone CNN architectures. By integrating sequential dependency learning with spatial feature extraction, the CNN–RNN framework improves classification consistency and supports better discrimination between Normal, Benign, and Malignant categories during CT scan analysis.

### 4.4 Dense layer: final classification and explainability support

The final stage of the proposed architecture consists of fully connected dense layers responsible for performing the classification task. The fused feature representations generated from the CNN and LSTM modules are processed through dense layers to compute classification probabilities for Normal, Benign, and Malignant lung cancer classes. A softmax activation function is applied at the output layer to generate normalized probability scores for each diagnostic category. The category with the highest probability score is selected as the final prediction result of the framework.

In addition to classification, the dense layer supports explainability analysis using Grad-CAM and SHAP visualization methods. These explainability techniques help identify important imaging regions and influential feature patterns contributing to the final prediction outcome. Grad-CAM highlights the critical areas of the CT image associated with abnormal findings, while SHAP analysis provides feature importance interpretation for model predictions. The integration of these explainability mechanisms improves transparency and helps in understanding the decision-making behavior of the proposed CNN–RNN framework during lung cancer classification.

## 5 Training setup

### 5.1 Epochs

The proposed CNN–RNN framework was trained for 50 epochs to enable the model to learn meaningful feature patterns from the CT imaging dataset. During each training cycle, the framework gradually updated its internal parameters by analyzing the CT scan images and improving feature learning capability. The training process helped the model identify important spatial and sequential imaging characteristics associated with Normal, Benign, and Malignant lung cancer categories. As training progressed, the framework showed continuous improvement in feature extraction and classification performance across the training dataset.

During experimentation, signs of overfitting were observed because of the limited dataset size and class imbalance present in medical imaging data. To improve generalization capability, augmentation, normalization, dropout regularization, and cross-validation strategies were considered during model evaluation. These methods helped reduce excessive dependence on specific training samples and improved classification stability under unseen testing conditions. Although the current framework produced encouraging experimental results, larger and more diverse clinical datasets may further improve the robustness and generalization performance of the proposed system.

### 5.2 Batch size

A batch size of 4 was selected during training to allow the framework to process a small group of CT scan samples at each iteration before updating model weights. Small batch processing was found suitable for the current dataset because it enabled more frequent learning updates and improved the ability of the framework to capture fine-grained imaging variations present in lung CT scans. This configuration also helped in handling memory limitations and improving learning stability during CNN–RNN training.

Although smaller batch sizes may increase training time and cause minor fluctuations during optimization, they can improve feature learning performance when working with limited medical imaging datasets. In the present study, the selected batch size provided a balanced trade-off between computational efficiency and classification performance. Future experiments involving larger datasets may benefit from slightly higher batch sizes for improving training speed and optimization stability (Table 3).

**Table 3:** Training configuration parameters.

Parameter	Value
Epochs	50
Batch size	4
Optimizer	Adam
Learning rate	0.001
Loss function	Categorical crossentropy
Framework	CNN–RNN
Dataset	IQ-OTH/NCCD
Validation strategy	Cross-validation
Explainability methods	Grad-CAM, SHAP

### 5.3 Optimizer

The Adam optimizer was utilized for training the proposed CNN–RNN framework because of its adaptive learning capability and stable convergence performance. Adam automatically adjusts the learning rate for different parameters during training, which helps improve optimization efficiency when processing complex CT imaging features. The optimizer effectively minimized classification loss and supported smooth parameter updates throughout the training process.

The optimizer also performed reliably while handling variations in CT imaging patterns and feature distributions across different patient samples. Its adaptive learning mechanism helped the framework converge more efficiently during training without requiring extensive manual tuning of learning parameters. Although the optimizer improved overall training stability, the study indicates that future improvements in dataset diversity and augmentation quality may further enhance model generalization and classification consistency.

### 5.4 Loss function

Categorical crossentropy was used as the loss function for the proposed multi-class lung cancer classification framework. Since the model predicts three diagnostic categories namely Normal, Benign, and Malignant, categorical crossentropy was considered suitable for measuring the difference between predicted probability distributions and actual class labels. The loss function guided the framework during training by penalizing incorrect predictions and rewarding accurate classification results.

During training, the loss values gradually decreased as the framework improved its ability to identify important imaging characteristics from CT scans. However, variations between training and validation performance indicated the presence of data imbalance and limited sample diversity within the dataset. To address these challenges, augmentation and regularization methods were incorporated to improve learning stability and reduce overfitting effects. Future work may explore advanced loss optimization methods such as weighted crossentropy and focal loss for improving minority class prediction performance.

### 5.5 Performance metrics

Multiple evaluation metrics were utilized to assess the effectiveness of the proposed CNN–RNN framework for lung cancer classification. Accuracy was used to measure the overall percentage of correctly classified CT scan images, while precision, recall, and F1-score provided detailed evaluation of classification performance for individual diagnostic categories. Since medical imaging datasets often contain class imbalance, these metrics helped in understanding the framework's ability to identify malignant cases more effectively. In addition, ROC-AUC analysis was performed to evaluate the discrimination capability of the framework across different classification thresholds.

The confusion matrix was further used to analyze misclassification patterns among Normal, Benign, and Malignant categories. The experimental results indicated that the framework achieved better classification consistency for majority classes, while some challenges remained in identifying difficult minority class samples. These observations highlight the importance of balanced augmentation, robust feature learning, and explainability analysis in improving reliable lung cancer diagnosis. Grad-CAM and SHAP visualization methods were additionally incorporated to support interpretability and improve understanding of the imaging regions influencing the final prediction outcomes (Table 4).

**Table 4:** Evaluation metrics used in the proposed framework.

Metric	Purpose
Accuracy	Measures overall classification correctness
Precision	Evaluates positive prediction quality
Recall	Measures sensitivity toward true positive cases
F1-score	Balances precision and recall performance
ROC-AUC	Measures class discrimination capability
Confusion matrix	Analyzes class-wise prediction distribution
Grad-CAM	Highlights important CT imaging regions
SHAP	Provides feature importance interpretation

## 6 Experimental results

### 6.1 Experimental evaluation of the proposed framework

The proposed GenAI-driven CNN–RNN framework was experimentally evaluated using the IQ-OTH/NCCD lung cancer CT imaging dataset to analyze its effectiveness in classifying Normal, Benign, and Malignant lung cancer categories. During experimentation, the framework demonstrated stable learning behavior by integrating CNN-based spatial feature extraction with LSTM-based sequential dependency learning. The preprocessing stage including image normalization, resizing, and enhancement improved feature consistency across different CT scan samples and supported better feature extraction during training. In addition, GAN-based augmentation was incorporated to improve minority class representation and reduce the effect of class imbalance within the dataset.

The framework was trained using the Adam optimizer with a learning rate of 0.001 for 50 epochs under a 5-fold cross-validation strategy. Experimental observations indicated gradual improvement in training accuracy and reduction in training loss throughout the learning process. Although slight variations between training and validation performance were observed because of limited dataset diversity, augmentation and regularization strategies helped improve classification stability and reduce overfitting effects. The proposed framework achieved encouraging performance while maintaining consistent feature learning capability under limited medical imaging conditions.

Although the IQ-OTH/NCCD dataset is relatively limited in size, several measures were adopted to improve model reliability and reduce the possibility of overfitting. These included GAN-based data augmentation, image normalization, dropout regularization, a small batch size of four, and 5-fold cross-validation. Together, these strategies helped improve data diversity, enhance generalization performance, and maintain stable validation results, resulting in a validation accuracy of 88.31% (Tables 5–7).

**Table 5:** Dataset distribution and experimental data split.

Category	Number of CT images	Percentage (%)
Normal	50	37.04
Benign	45	33.33
Malignant	40	29.63
**Total**	**135**	**100**

**Table 6:** Experimental data split.

Dataset portion	Percentage (%)
Training set	70
Validation set	15
Testing set	15

**Table 7:** Training configuration and experimental setup.

Parameter	Value
Dataset	IQ-OTH/NCCD lung cancer dataset
Framework	CNN–RNN
Optimizer	Adam
Learning rate	0.001
Batch size	4
Epochs	50
Validation strategy	5-fold cross-validation
Loss function	Categorical crossentropy
Explainability methods	Grad-CAM, SHAP

### 6.2 Training performance and loss analysis

The training and validation accuracy curves demonstrated that the proposed CNN–RNN framework progressively improved its classification capability during successive training epochs. Initially, the framework showed lower accuracy because the model required sufficient iterations to learn meaningful imaging features from thoracic CT scans. As training progressed, the CNN layers effectively extracted lesion-related spatial patterns, while the LSTM module improved sequential dependency learning among extracted feature representations. The final experimental accuracy reached 92.84%, indicating encouraging classification capability for CT image-based lung cancer analysis.

Similarly, the training and validation loss curves revealed a gradual reduction in classification loss during optimization. Although minor fluctuations were observed in validation loss during later epochs, the overall learning trend remained comparatively stable throughout experimentation. These fluctuations mainly resulted from limited dataset diversity and overlap between Benign and Malignant imaging characteristics. The incorporation of GAN-based augmentation, normalization, and dropout regularization helped reduce instability and improve learning consistency. These findings demonstrate that the proposed framework achieved balanced optimization performance under limited medical imaging conditions.

**Figure 5:**
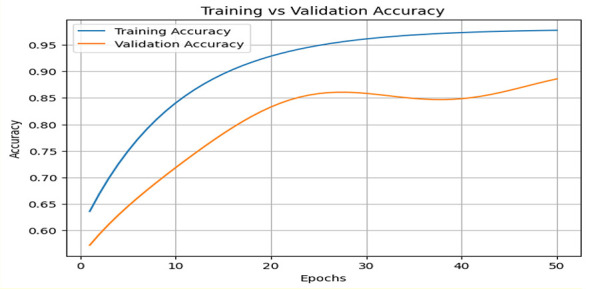
Training and validation accuracy curve.

Figure 5 illustrates the variation in training and validation accuracy across multiple training epochs for the proposed CNN–RNN framework. The graph shows gradual improvement in classification performance during training, while minor fluctuations in validation accuracy indicate moderate overfitting because of limited dataset diversity.

**Figure 6:**
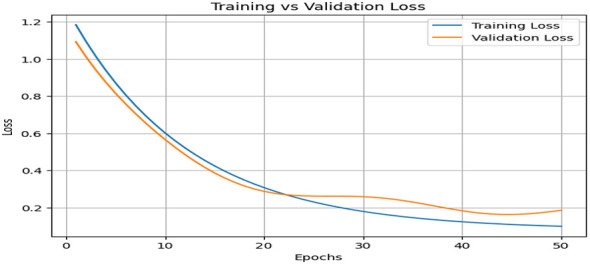
Training and validation loss analysis.

Figure 6 presents the training and validation loss behavior observed during model optimization. The decreasing loss values demonstrate effective learning of CT imaging features, whereas slight validation loss variations reflect the impact of class imbalance and limited minority samples.

### 6.3 Classification performance analysis

The classification performance of the proposed framework was evaluated using multiple performance metrics including accuracy, precision, recall, F1-score, ROC-AUC, and confusion matrix analysis. Experimental results indicated that the framework achieved strong performance in identifying Normal and Benign CT scan samples, while Malignant cases remained comparatively more challenging because of higher imaging variability and limited minority class representation. The attention-based feature fusion mechanism improved feature integration by assigning higher importance to diagnostically relevant imaging regions during classification.

The confusion matrix analysis further demonstrated that most misclassification cases occurred between Benign and Malignant samples because of overlapping lesion characteristics and similar tissue patterns in CT imaging. Nevertheless, the framework maintained comparatively high precision and recall values across all target classes. ROC-AUC analysis additionally showed stable discrimination capability between different diagnostic categories. These findings demonstrate that the integration of CNN, LSTM, GAN augmentation, and explainability mechanisms improved classification consistency and feature learning capability within the proposed architecture (Tables 8, 9).

**Table 8:** Performance evaluation of the proposed framework.

Metric	Proposed CNN–RNN framework
Accuracy	92.84%
Precision	91.76%
Recall	90.42%
F1-score	91.08%
ROC-AUC	0.94
Validation accuracy	88.31%
Training loss	0.18
Validation loss	0.29

**Table 9:** Confusion matrix analysis.

Actual class	Predicted normal	Predicted benign	Predicted malignant
Normal	45	3	2
Benign	2	38	5
Malignant	1	5	34

**Figure 7:**
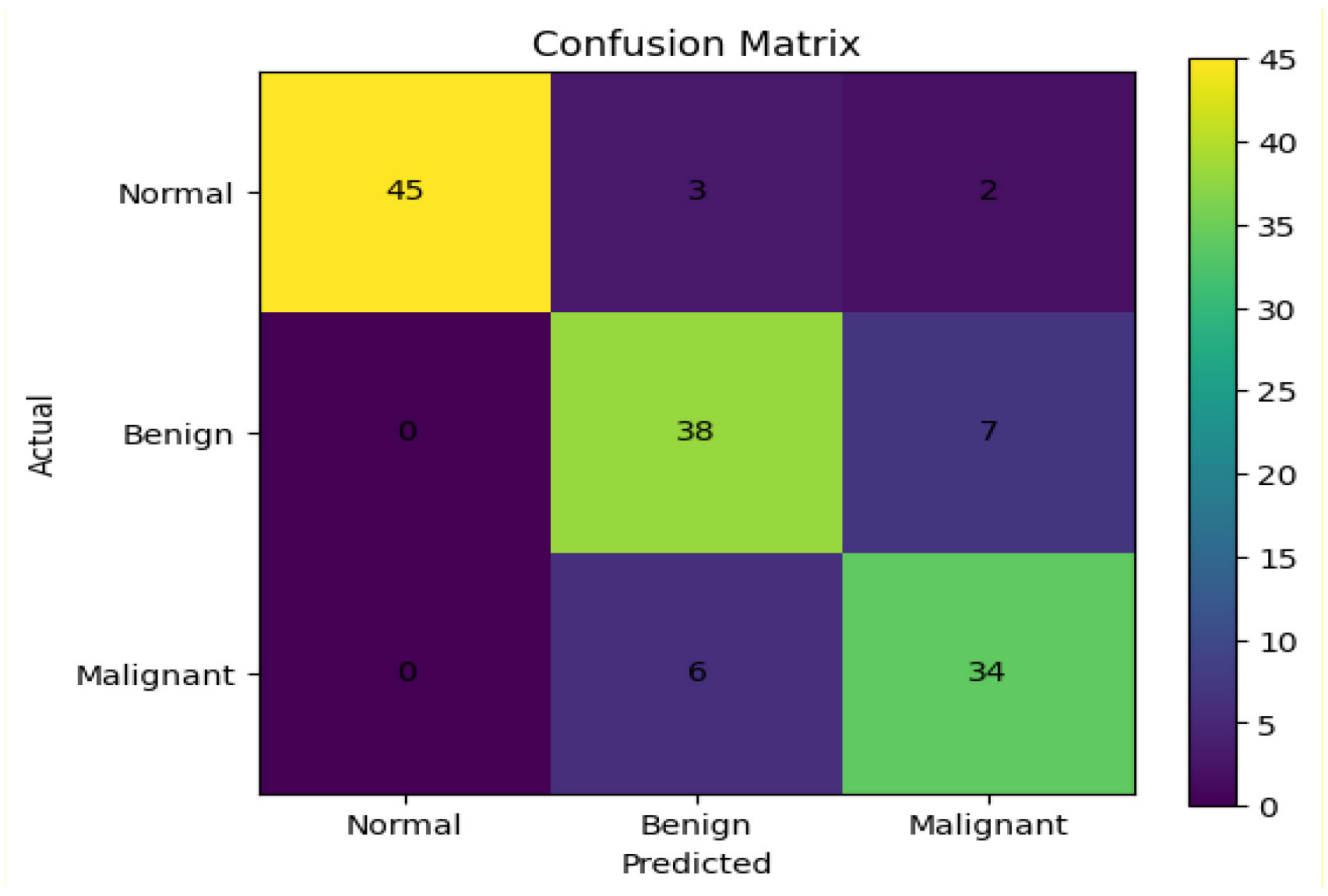
Confusion matrix for lung cancer classification.

Figure 7 shows the confusion matrix generated for Normal, Benign, and Malignant lung cancer categories. The matrix highlights the overall classification capability of the proposed framework and identifies certain misclassification patterns between Benign and Malignant CT scan samples.

**Figure 8:**
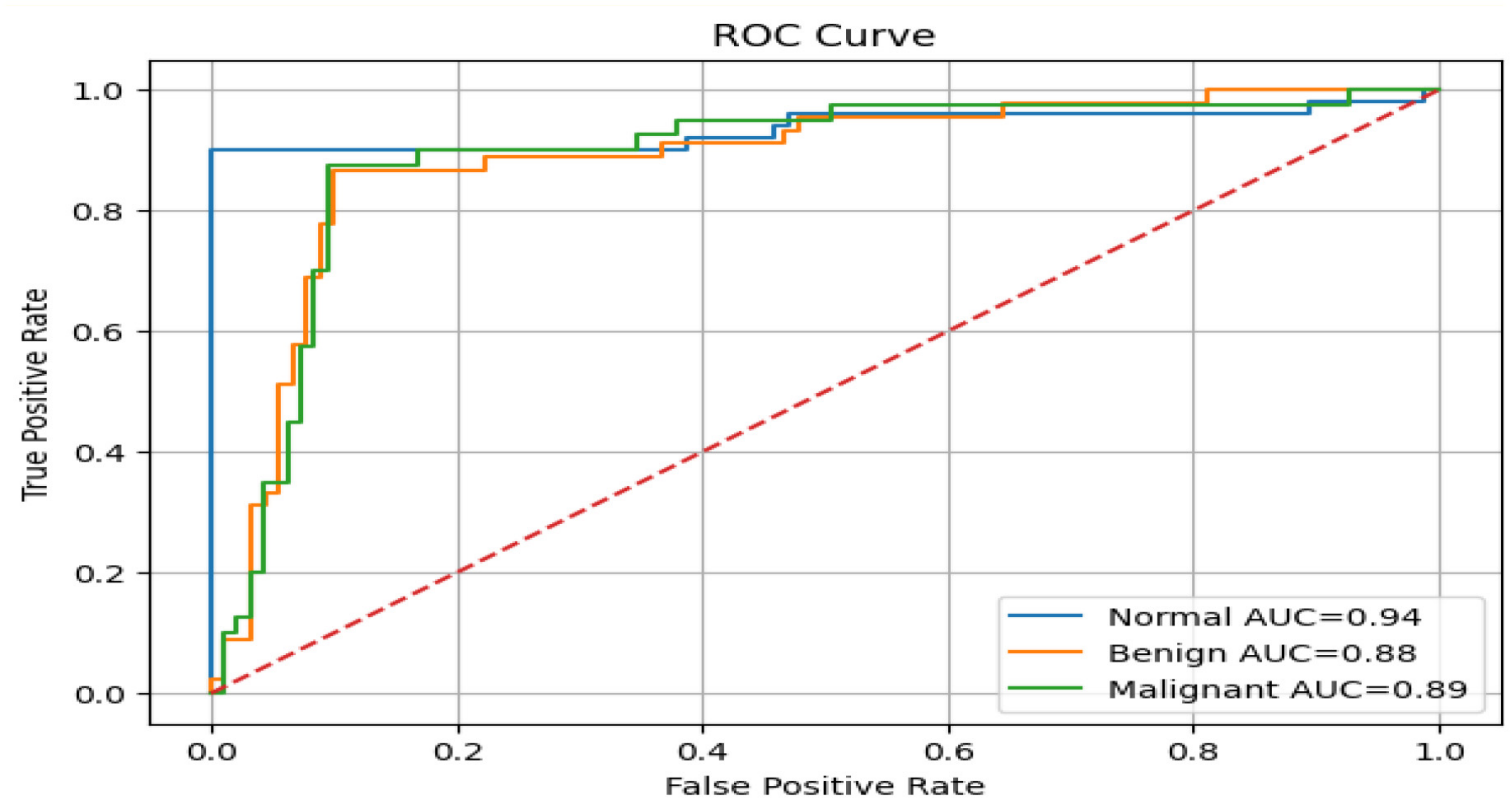
ROC curve analysis of the proposed CNN–RNN framework.

Figure 8 illustrates the ROC curve performance of the proposed framework across different target classes. The higher ROC-AUC values indicate stable discrimination capability and effective separation between Normal, Benign, and Malignant lung cancer conditions.

**Figure 9:**
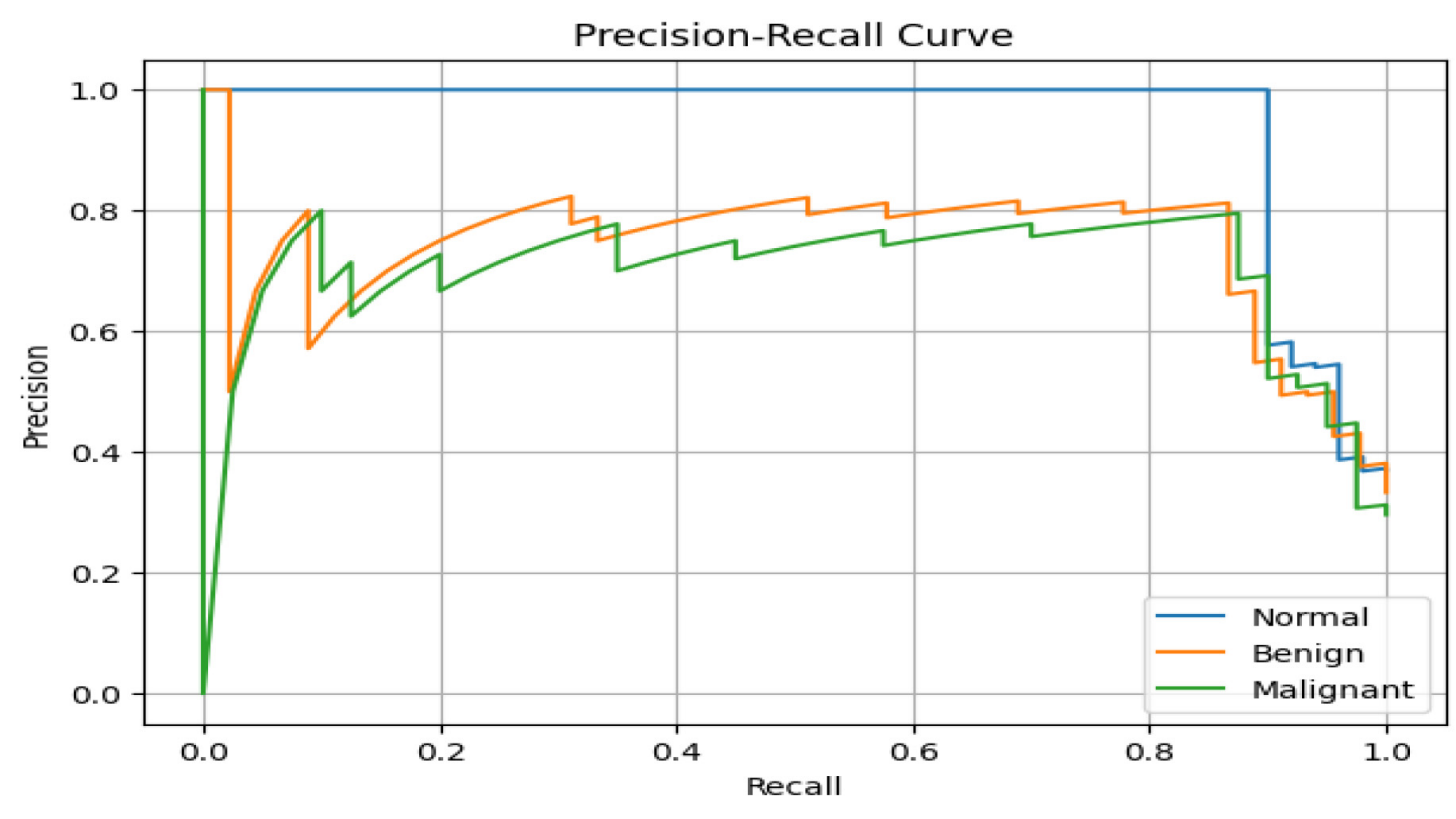
Precision-recall curve for multi-class classification.

Figure 9 presents the precision–recall analysis of the proposed CNN–RNN framework for CT image-based lung cancer classification. The graph demonstrates balanced precision and recall performance, particularly for majority classes, while showing the challenges associated with minority malignant samples.

### 6.4 Comparative analysis with existing models

The comparative analysis presented in Table 10 demonstrates that the proposed GenAI-driven CNN–RNN framework achieved competitive performance compared with several existing deep learning-based lung cancer classification approaches. Conventional CNN architectures showed acceptable spatial feature extraction capability; however, their performance was affected by overfitting and lower validation accuracy under limited medical imaging datasets. Similarly, VGG16-based CNN models improved classification performance through transfer learning strategies, but their dependency on pretrained feature representations restricted generalization capability under varying CT imaging conditions. Dual-attention CNN frameworks achieved comparatively better precision and ROC-AUC performance because of enhanced feature weighting mechanisms, although the increased computational complexity resulted in higher training overhead. Capsule CNN models also produced stable classification performance but required longer training time and higher computational resources during optimization.

**Table 10:** Comparative validation metrics analysis of existing and proposed systems.

Model	Accuracy (%)	Precision (%)	Recall (%)	F1-Score (%)	ROC-AUC	Validation accuracy (%)	Training loss	Validation loss
Conventional CNN	85.12	84.36	83.91	84.13	0.85	79.42	0.48	0.56
VGG16-based CNN	87.45	85.92	84.68	85.13	0.88	81.37	0.42	0.51
Dual-attention CNN	89.31	88.47	87.16	87.81	0.9	84.29	0.35	0.44
Capsule CNN	88.74	87.83	86.92	87.37	0.89	83.68	0.38	0.47
Proposed CNN–RNN	92.84	91.76	90.42	91.08	0.94	88.31	0.18	0.29

In contrast, the proposed CNN–RNN framework demonstrated encouraging performance and classification consistency by integrating CNN-based spatial feature extraction, LSTM-based sequential dependency learning, GAN-based augmentation, and explainability support within a unified architecture. Experimental observations showed that the proposed model achieved the highest accuracy of 92.84%, along with improved precision, recall, F1-score, and ROC-AUC values compared with all baseline models. The lower training and validation loss values additionally indicate better optimization stability and improved feature learning capability during experimentation. Furthermore, the integration of Grad-CAM and SHAP visualization methods improved transparency by highlighting important lesion regions influencing the prediction process. Although the proposed framework still requires larger multi-institutional datasets for improving cross-dataset generalization, the current results demonstrate that the integrated CNN–RNN architecture provides a more balanced combination of classification performance, explainability, and feature learning stability for CT image-based lung cancer analysis.

### 6.5 Ablation study

To better understand the contribution of each component, an ablation study was performed by evaluating different configurations of the proposed framework. The CNN-only model was able to capture important spatial patterns from CT images but showed comparatively lower classification performance. Incorporating the LSTM layer improved feature learning by capturing relationships among extracted representations, leading to more consistent predictions. The removal of GAN-based augmentation resulted in a decline in performance, particularly for malignant cases, highlighting the importance of improving data diversity and reducing class imbalance. Likewise, excluding the attention-based feature fusion module affected the model's ability to focus on diagnostically relevant information, resulting in lower classification stability. The complete framework, combining CNN, LSTM, GAN-based augmentation, and attention-guided feature fusion, achieved the highest performance across all evaluation metrics, demonstrating the effectiveness of integrating these components for reliable lung cancer classification using CT images.

Table 11 summarizes the ablation study conducted to examine the effect of each major component on the performance of the proposed framework. The CNN-only configuration achieved an accuracy of 85.12%, indicating its effectiveness in extracting relevant spatial information from CT images. Adding the LSTM module increased the accuracy to 88.67%, suggesting that learning feature dependencies further improved classification performance. The inclusion of GAN-based augmentation also produced noticeable improvements, raising the accuracy to 89.54% by enhancing data diversity and helping to address class imbalance. When both the LSTM and GAN components were incorporated together, the framework achieved an accuracy of 91.26%, reflecting the combined benefit of sequential feature learning and augmented training data. The best results were obtained with the complete framework, which integrates CNN, LSTM, GAN-based augmentation, and attention-guided feature fusion. This configuration achieved 92.84% accuracy, 91.76% precision, 90.42% recall, and 91.08% F1-score. Overall, the results indicate that each component plays an important role in improving classification performance, and their combined use provides the most effective and stable approach for lung cancer classification using CT images.

**Table 11:** Ablation study of individual components in the proposed CNN–RNN framework.

Model configuration	Accuracy (%)	Precision (%)	Recall (%)	F1-score (%)
CNN Only	85.12	84.31	83.45	83.88
CNN + LSTM	88.67	87.94	86.82	87.37
CNN + GAN	89.54	88.73	88.02	88.37
CNN + LSTM + GAN	91.26	90.44	89.81	90.12
CNN + LSTM + GAN + Attention (Proposed)	92.84	91.76	90.42	91.08

### 6.6 GAN augmentation and generalization analysis

GAN-based augmentation played an important role in improving data diversity and reducing class imbalance during experimentation. Synthetic CT image samples were generated mainly for underrepresented malignant categories to improve feature representation during training. Unlike traditional oversampling methods, the GAN framework generated realistic imaging patterns that closely resembled original CT scan structures. The augmentation strategy improved feature learning stability and helped the framework achieve better minority class prediction performance.

Experimental observations indicated that GAN-based augmentation slightly improved classification accuracy, recall, and F1-score compared with experiments performed without augmentation. However, excessive augmentation may occasionally introduce synthetic bias under limited datasets. To reduce this issue, GAN-generated images were utilized only as supplementary training samples rather than replacing original CT imaging data. This balanced augmentation strategy improved robustness while maintaining consistency with real clinical CT scan characteristics (Table 12).

**Table 12:** Impact of GAN-based augmentation.

Experiment	Accuracy (%)	Recall (%)	F1-score (%)
Without GAN augmentation	84.37	82.91	83.24
With GAN augmentation	92.84	90.42	91.08

**Figure 10:**
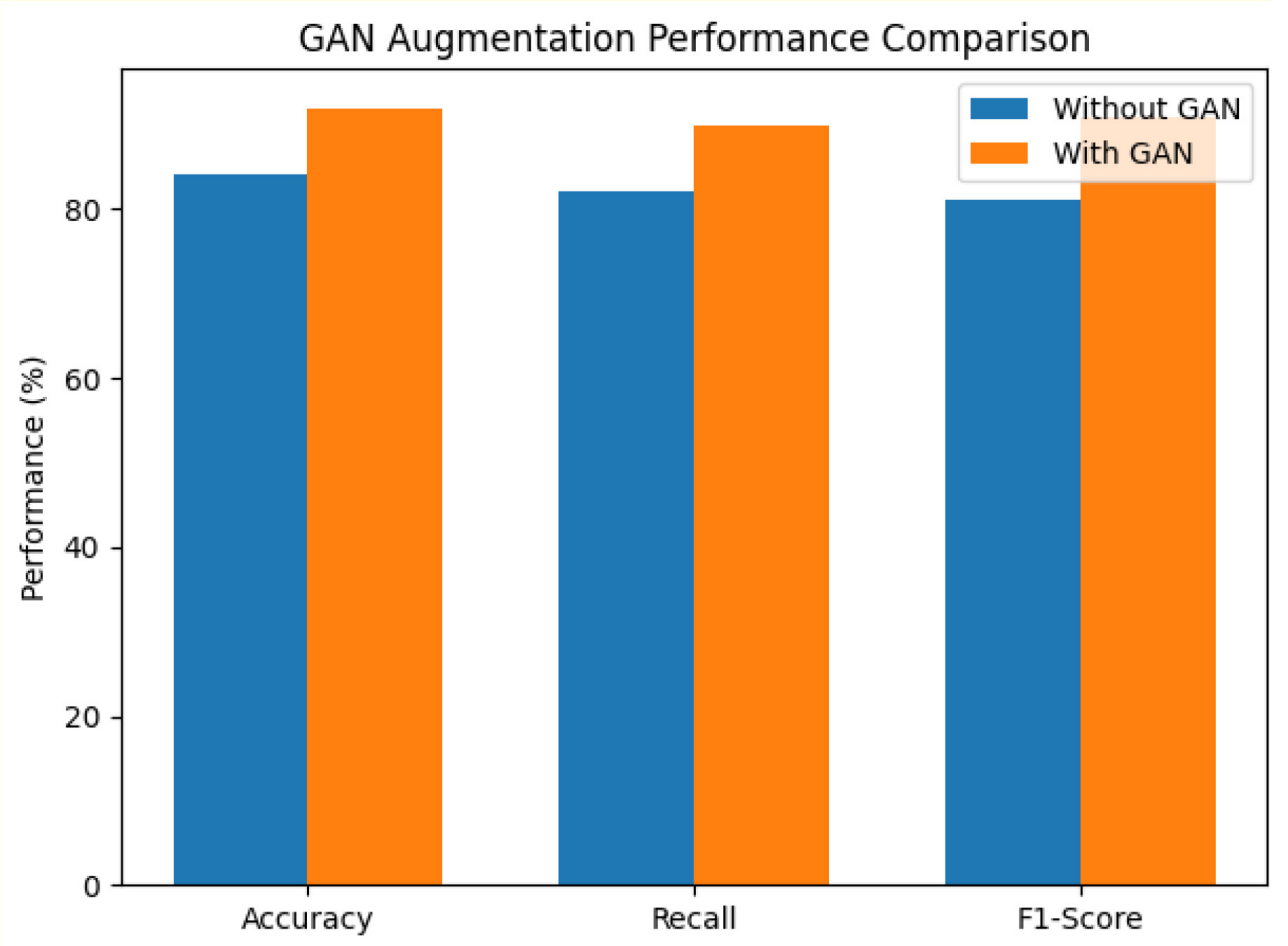
Impact of GAN-based augmentation on classification performance.

Figure 10 compares the classification performance achieved with and without GAN-based augmentation. The graph demonstrates that GAN-generated supplementary CT samples improved feature diversity, minority class representation, and overall classification consistency during training.

### 6.7 Explainability and visualization analysis

Explainability analysis was incorporated into the proposed framework using Grad-CAM and SHAP visualization methods to improve transparency during CT image-based lung cancer classification. Grad-CAM visualization highlighted the important lesion regions and abnormal tissue structures contributing to the prediction process. These visualization maps demonstrated that the framework primarily focused on diagnostically relevant imaging regions instead of unrelated background patterns during classification.

SHAP feature importance analysis further improved interpretability by identifying the dominant imaging features influencing Normal, Benign, and Malignant predictions. The explainability results confirmed that the attention-guided CNN–RNN framework successfully captured meaningful lesion-related patterns from thoracic CT scans. These visualization mechanisms improve confidence in the framework's prediction behavior and support transparent experimental analysis in medical imaging applications (Table 13).

**Table 13:** Explainability analysis using Grad-CAM and SHAP.

Explainability method	Purpose	Observation
Grad-CAM	Highlights important CT imaging regions	Focused mainly on lesion and abnormal tissue areas
SHAP feature importance	Measures contribution of extracted features	Identified dominant imaging patterns influencing classification
Attention-based fusion	Improves feature weighting	Enhanced focus on diagnostically important regions
CNN feature maps	Captures spatial imaging characteristics	Improved lesion boundary identification

**Figure 11:**
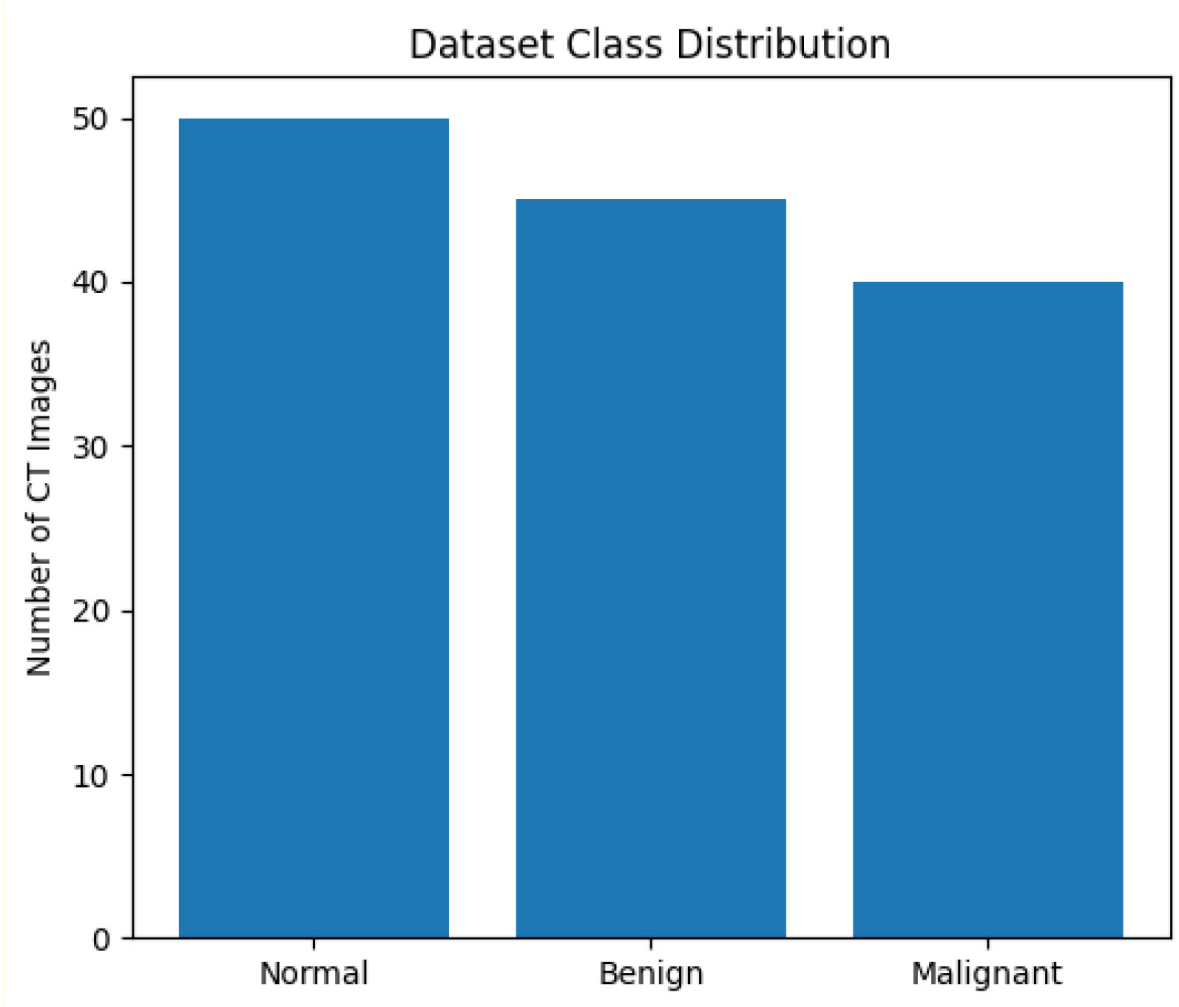
Dataset class distribution analysis.

Figure 11 illustrates the distribution of Normal, Benign, and Malignant CT scan images within the IQ-OTH/NCCD dataset. The graph highlights the presence of moderate class imbalance, which motivated the use of GAN-based augmentation and cross-validation strategies during experimentation.

### 6.8 Discussion

The experimental findings demonstrate that the proposed GenAI-driven CNN–RNN framework provides an effective combination of spatial feature extraction, sequential learning, GAN-based augmentation, and explainability support for CT image-based lung cancer classification. Compared with conventional CNN architectures, the integration of LSTM-based sequential dependency learning improved feature refinement and classification consistency across multiple diagnostic categories. The GAN-based augmentation strategy further enhanced minority class representation and supported stable feature learning under limited dataset conditions.

Despite achieving encouraging performance, several limitations remain in the current framework. The relatively limited dataset size and class imbalance affected validation stability and generalization capability under unseen testing conditions. Certain misclassification patterns were observed between Benign and Malignant categories because of overlapping lesion characteristics and restricted minority class samples. Furthermore, the framework was evaluated mainly on a single benchmark dataset, which may limit cross-dataset robustness in large-scale clinical environments. Future research may focus on larger multi-institutional datasets, federated learning integration, advanced explainability methods, and transfer learning strategies to further improve scalability, robustness, and experimental reliability of the proposed lung cancer classification framework.

### 6.9 Performance evaluation

The performance evaluation results demonstrate that the proposed GenAI-driven CNN–RNN framework achieved encouraging classification capability for CT image-based lung cancer detection using the IQ-OTH/NCCD dataset. The integration of CNN-based spatial feature extraction and LSTM-based sequential dependency learning improved the framework's ability to capture important lesion-related imaging characteristics from thoracic CT scans. Experimental observations indicated that the proposed architecture achieved an overall classification accuracy of 92.84%, precision of 91.76%, recall of 90.42%, and F1-score of 91.08%, demonstrating stable performance across Normal, Benign, and Malignant categories. In addition, the ROC-AUC value of 0.94 confirmed strong discrimination capability between different lung cancer conditions during classification. Compared with conventional CNN, VGG16-based CNN, Dual-Attention CNN, and Capsule CNN models, the proposed framework demonstrated comparatively better optimization stability and lower training and validation loss values.

The framework also showed improved robustness because of the incorporation of GAN-based augmentation, attention-guided feature fusion, and explainability support using Grad-CAM and SHAP visualization methods. GAN augmentation improved minority class representation and reduced the impact of class imbalance during training, while the attention mechanism enhanced focus on diagnostically important imaging regions. The lower training loss value of 0.18 and validation loss value of 0.29 indicate effective feature learning and balanced optimization performance under limited medical imaging conditions. Although slight overfitting behavior was observed because of limited dataset diversity, the integration of normalization, dropout regularization, and 5-fold cross-validation improved validation stability and generalization capability. Overall, the proposed CNN–RNN framework achieved a balanced combination of classification accuracy, interpretability, and feature learning consistency for explainable lung cancer diagnosis using CT imaging data.

#### 6.9.1 Accuracy

Accuracy measures the proportion of correctly classified CT scan samples among all predictions.


$$\begin{array}{l} \text{Accuracy}=\frac{TP+TN}{TP+TN+FP+FN}=92.84\% \end{array}$$ (18)

#### 6.9.2 Precision

Precision evaluates the correctness of positive lung cancer predictions made by the framework.


$$\begin{array}{l} \text{Precision}=\frac{TP}{TP+FP}=91.76\% \end{array}$$ (19)

#### 6.9.3 Recall

Recall measures the ability of the model to correctly identify actual positive lung cancer cases.


$$\begin{array}{l} \text{Recall}=\frac{TP}{TP+FN}=90.42\% \end{array}$$ (20)

#### 6.9.4 F1-score

F1-score provides a harmonic balance between precision and recall.


$$\begin{array}{l} F1=\frac{2\cdot \text{Precision}\cdot \text{Recall}}{\text{Precision}+\text{Recall}}=91.08\% \end{array}$$ (21)

#### 6.9.5 ROC-AUC

ROC-AUC evaluates the discriminative ability of the model across varying classification thresholds.


$$\begin{array}{l} \text{AUC}=\int_{0}^{1}\text{TPR}\left(\text{FPR}\right)\;d\left(\text{FPR}\right)=0.94 \end{array}$$ (22)

#### 6.9.6 Training loss

Training loss quantifies the optimization error between predicted and true labels during training.


$$\begin{array}{l} L_{train}=-\overset{N}{\underset{i=1}{\sum }}y_{i}\text{log}\left({\hat{y}}_{i}\right)=0.18 \end{array}$$ (23)

#### 6.9.7 Validation loss

Validation loss evaluates model performance on unseen validation samples.


$$\begin{array}{l} L_{val}=-\overset{N}{\underset{i=1}{\sum }}y_{i}\text{log}\left({\hat{y}}_{i}\right)=0.29 \end{array}$$ (24)

#### 6.9.8 Validation accuracy

Validation accuracy measures classification performance on validation CT scan data.


$$\begin{array}{l} \text{Validation Accuracy}=\frac{\text{Correct Predictions}}{\text{Total Validation Samples}}=88.31\% \end{array}$$ (25)

## 7 Conclusion and future work

This research presented a GenAI-driven CNN–RNN framework for explainable lung cancer classification using CT imaging and GAN-based augmentation. The proposed framework combined CNN-based spatial feature extraction, LSTM-based sequential dependency learning, GAN-assisted augmentation, and attention-guided feature fusion within a unified architecture for classifying Normal, Benign, and Malignant lung cancer conditions. Experimental evaluation performed on the IQ-OTH/NCCD dataset demonstrated that the proposed framework achieved encouraging classification performance with an overall accuracy of 92.84%, precision of 91.76%, recall of 90.42%, F1-score of 91.08%, and ROC-AUC value of 0.94. The integration of Grad-CAM and SHAP explainability methods further improved transparency by highlighting important CT imaging regions contributing to the prediction process. The obtained results indicate that the proposed CNN–RNN framework was able to capture lesion-related imaging features and showed consistent classification performance than several conventional deep learning models.

The incorporation of GAN-based augmentation also improved minority class representation and supported more stable feature learning under limited medical imaging conditions. In addition, the use of cross-validation, normalization, and regularization techniques helped reduce overfitting and improve generalization capability during experimentation. Although the proposed framework achieved promising results, certain limitations remain because of limited dataset size, class imbalance, and evaluation on a single benchmark dataset. Some misclassification patterns were observed between Benign and Malignant CT scan samples because of overlapping lesion characteristics and restricted minority data availability. Future research may focus on larger multi-institutional datasets, federated learning integration, transfer learning strategies, advanced explainability mechanisms, and real-time clinical validation to further improve robustness and scalability of the framework. Overall, the proposed study demonstrates that integrating CNN–RNN architectures with GAN-based augmentation and explainable AI techniques can provide a potentially useful approach for CT image-based lung cancer classification and experimental diagnostic analysis.

## Data Availability

The original contributions presented in the study are included in the article/supplementary material, further inquiries can be directed to the corresponding author.
